# Climate Change and Human Health

**DOI:** 10.3390/ijerph110707347

**Published:** 2014-07-18

**Authors:** Jan C. Semenza

**Affiliations:** Office of the Chief Scientist, European Centre for Disease Prevention and Control, Tomtebodavagen 11A, SE-171 83 Stockholm, Sweden; E-Mail: Jan.Semenza@ecdc.europa.eu; Tel: +46-8-5860-1217; Fax: +46-8-5860-1296

Climate change impacts on human health span the trajectory of time—past, present, and future. The key finding from the Working Group II, Fifth Assessment Report (AR5) of the Intergovernmental Panel on Climate Change (IPCC) states that health impacts due to climate change have already occurred in the past, are currently occurring and will continue to occur, at least for the foreseeable future, even with immediate reductions in greenhouse gas emissions [1]. According to the IPCC, there has been increased heat-related mortality and decreased cold-related mortality in some regions as a result of warming (Box 1). Moreover, local changes in temperature and rainfall have altered the distribution of some water-borne illnesses and disease vectors. Impacts of climate-related extremes include alteration of ecosystems, disruption of food production and water supply, damage to infrastructure and settlements, morbidity and mortality, and consequences for mental health and human well-being [1]. 

In this special issue of the *International Journal of Environmental Research and Public Health* (*IJERPH*) we present 20 papers that add further weight to the conclusions of the AR5 of IPCC. Grouped in somewhat overlapping categories, three papers assess infectious disease threats from climate change, seven papers heat-related health impacts, one paper ozone-related mortality and nine papers discuss different aspects of adaptation (Box 2). 

## Infectious Diseases

Extreme weather events, such as heavy precipitation, are one of the hallmarks of global climate change. Runoff from such precipitation events can result in microbial transport and contamination of coastal water with implications to public health [2,3]. A paper in this special issue of the *IJERPH* examines these climatic events on beach closures due to high pathogen concentrations in recreational waters and the association with gastrointestinal-related hospital admissions [4]. Extreme rain events occurring the previous day were significantly associated with beach closures but not with GI-related hospital admissions. Coastal areas are also at risk for importation of competent disease vectors through cargo shipment. Freight containers (containing used tires or ornamental plants) can harbour the Asian tiger mosquito (*Aedes albopictus*) which is a vector for dengue and chikungunya. A paper by Thomas *et al*., in this issue describes freight container imports into areas in Europe that are climatically suitable for the vector *Ae. albopictus* [5]. The authors model the quantity of cargo imports from areas where the mosquito is endemic into harbours and further dissemination through inland waterways; current and future climatic suitability of the destination area are overlaid to model the long-term establishment of the mosquito.

Box 1 **IPCC, Fifth Assessment Report (AR5) key findings for human health, 2014.**

**Human health**

Until mid-century, projected climate change will impact human health mainly by exacerbating health problems that already exist (very high confidence). Throughout the 21st century, climate change is expected to lead to increases in ill-health in many regions and especially in developing countries with low income, as compared to a baseline without climate change (high confidence). Examples include greater likelihood of injury, disease, and death due to more intense heat waves and fires (very high confidence); increased likelihood of under-nutrition resulting from diminished food production in poor regions (high confidence); risks from lost work capacity and reduced labor productivity in vulnerable populations; and increased risks from food- and water-borne diseases (very high confidence) and vector-borne diseases (medium confidence). Positive effects are expected to include modest reductions in cold related mortality and morbidity in some areas due to fewer cold extremes (low confidence), geographical shifts in food production (medium confidence), and reduced capacity of vectors to transmit some diseases. But globally over the 21st century, the magnitude and severity of negative impacts are projected to increasingly outweigh positive impacts (high confidence). The most effective vulnerability reduction measures for health in the near-term are programs that implement and improve basic public health measures such as provision of clean water and sanitation, secure essential health care including vaccination and child health services, increase capacity for disaster preparedness and response, and alleviate poverty (very high confidence). By 2100 for the high-emission scenario RCP8.5, the combination of high temperature and humidity in some areas for parts of the year is projected to compromise normal human activities, including growing food or working outdoors (high confidence).

Source: [1]

Global environmental change has obviously implications for the distribution of infectious diseases in Europe [6] and regions with higher adaptive capacities will be able to counteract any climate change impacts from infectious diseases and be less vulnerable than areas with lower adaptive capacities [7]. This theme also emerges from the IPCC AR5 that health impacts from climate change should take into account underlying vulnerabilities that can potentially be mitigated or exacerbated by the socioeconomic context [1].

## Heat-Related and Ozone Health Impacts

**Figure 1:**
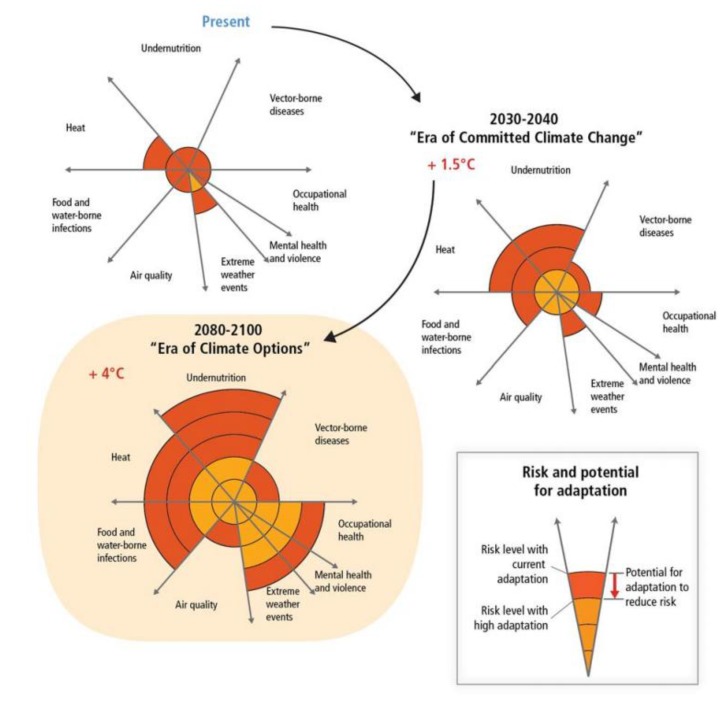
Conceptual presentation of the health impacts from climate change and the potential for impact reduction through adaptation (Source: [1]).

Note: Impacts are identified in eight health-related sectors based on assessment of the literature and expert judgments by authors of Chapter 11 of IPCC WGII AR5 [1]. The width of the slices indicates in a qualitative way the relative importance in terms of burden of ill-health globally at present and should not be considered completely independent. Impact levels are presented for the near-term era of committed climate change (2030–2040), in which projected levels of global mean temperature increases do not diverge substantially across emissions scenarios. For some sectors, e.g., vector-borne diseases, heat/cold stress, and agricultural production and under-nutrition, there may be benefits to health in some areas, but the net impact is expected to be negative. Estimated impacts are also presented for the longer-term era of climate options (2080–2100), for global mean temperature increase of 4 °C above preindustrial levels, which could potentially be avoided by vigorous mitigation efforts taken soon. For each timeframe, impact levels are estimated for the current state of adaptation and for a hypothetical highly adapted state, indicated by different colours.

A striking and worrying finding from AR5 is that the capacity of societal structures and natural systems to adapt is finite, and thus subject to adaptation limits (Figure 1) [1]. For example, coastal cities around the world have already adapted to 19 cm of sea level rise, but some coastal cities might need to adapt to 98 cm as the projections indicate. In many instances adapting to such a differential might not be possible.

Box 2 **Paper summary in special issue “Climate Change and Human Health”.**

**Infectious diseases **

- Article: Extreme Precipitation and Beach Closures in the Great Lakes Region: Evaluating Risk among the Elderly
- Article: Implementing Cargo Movement into Climate Based Risk Assessment of Vector-Borne Diseases
- Article: Indicators for Tracking European Vulnerabilities to the Risks of Infectious Disease Transmission due to Climate Change

**Heat-related health impacts **

- Article: Heat-Related Deaths in Hot Cities: Estimates of Human Tolerance to High Temperature Thresholds (H)
- Article: Effect of Ambient Temperature on Australian Northern Territory Public Hospital Admissions for Cardiovascular Disease among Indigenous and Non-Indigenous Populations (H)
- Article: Comparison of UTCI with Other Thermal Indices in the Assessment of Heat and Cold Effects on Cardiovascular Mortality in the Czech Republic (H)
- Article: Projected Heat-Related Mortality in the U.S. Urban Northeast (H)
- Article: Risk Factors, Health Effects and Behaviour in Older People during Extreme Heat: A Survey in South Australia (H)
- Article: Extreme Heat and Health: Perspectives from Health Service Providers in Rural and Remote Communities in South Australia (H)
- Article: A Cross-Sectional, Randomized Cluster Sample Survey of Household Vulnerability to Extreme Heat among Slum Dwellers in Ahmedabad, India (H)

**Ozone**

- Article: The Impact of Climate Change on Ozone-Related Mortality in Sydney

**Adaptation**

- Article: European Monitoring Systems and Data for Assessing Environmental and Climate Impacts on Human Infectious Diseases (A)
- Article: Data Mashups: Potential Contribution to Decision Support on Climate Change and Health (A)
- Article: Using Social Network Analysis to Evaluate Health-Related Adaptation Decision-Making in Cambodia (A)
- Article: Health in the New Scenarios for Climate Change Research (A)
- Review: A Review of National-Level Adaptation Planning with Regards to the Risks Posed by Climate Change on Infectious Diseases in 14 OECD Nations (A)
- Review: Impediments to Comprehensive Research on Climate Change and Health (A)
- Article: Focusing Resource Allocation-Wellbeing as a Tool for Prioritizing Interventions for Communities at Risk (A)
- Article: Building Resilience against Climate Effects—A Novel Framework to Facilitate Climate Readiness in Public Health Agencies
- Article: Strategies to Reduce the Harmful Effects of Extreme Heat Events: A Four-City Study (H)

An important contribution in this special issue of *IJERPH* addresses the limitation of humans to adapt to high ambient temperatures. A paper by Harlan *et al*. analyses heat-related mortality in cities with a hot climate at present. Even in cities that have already adapted to predictable periods of high temperatures, heat-related mortality increases during hot spells [8]. There are obviously physiological limits to extreme heat exposure and global climate change will turn certain parts of the world that are currently highly populated uninhabitable if the average global temperature rises by at least 7 °C [9]. Petkova *et al*. project the anticipated impact of heat-related mortality in the three largest cities of the Northeast of the United States (Boston, New York City and Philadelphia) using the climate models of AR5 [10]. The projected heat-related mortality rates in the 2020s, 2050s and 2080s were the highest in New York city, followed by Philadelphia and Boston revealing considerable vulnerabilities and challenges to adaptation. 

A number of papers in this issue describe specific populations that are vulnerable to heat-related mortality and morbidity. Indigenous, rural and elderly individuals in Australia and urban slum dwellers in India are particularly at risk [11,12,13,14]. The impact of climate change on ozone-related mortality at the local scale across Sydney, Australia was examined and projected to cause an additional 55 to 65 deaths (above current levels) in the decade 2051–2060 [15].

## Adaptation

A paper by Bowen *et al*. uses social network analysis to identify key organisational stakeholders involved in adaptation activities, such as government ministries in Cambodia; in contrast, donor agencies, development banks and non-government organisations contribute much less to adaptation strategies [16]. Adaptive capacity to extreme weather conditions and infectious diseases depends on effective governance and access to resources. Adaptation strategies for populations most vulnerable to the harmful effects of extreme heat in different settings are discussed in a paper by White-Newsome *et al*. [17]. A paper by Marinucci *et al*. in this issue presents a framework to systematically use climate projections to guide public health adaptation. The strategy integrates assessments of climate change impacts, vulnerability assessments, modelling of projected health impacts, evidence-based evaluation of intervention options, strategy for implementing interventions, and systematic evaluation of all activities in an iterative framework [18]. Since socioeconomic status determines adaptive capacity, global pathways have to be developed to demarcate different options of adaptation to be taken. Kris Ebi describes the implications of five reference socioeconomic development pathways to human health, as they relate to increasing socioeconomic and environmental challenges to adaptation and mitigation [19]. These pathways are important to project the adaptive capacity into the future. Moreover, the systematic collection, merging, integration and analysis of environmental and climatic data with epidemiologic data can generate forecasts and predictions of health outcomes that can help to promote resilience to climate change; two papers in this issue discuss the use of such data sources for epidemiologic analyses for future threat evaluations, outbreak management and interventions to reduce disease burden [20,21]. However, these advanced modelling techniques have their limitations too. Anthony McMichael elaborates on the difficulties of conducting global environmental change research: there is a need to examine climate change sensitivity of population health and social stability of past societies; expand the research methods beyond traditional boundaries, and advance trans-disciplinary approaches to forecasting health risk to populations [22]. Thus, even with the contributions in this special issue of *IJERPH*, there are still considerable technical hurdles and obstacles to be taken, not to mention the political ones, in order to advance the field and minimize human health impacts from climate change.
